# Dental treatment under general anesthesia in a group of patients with cerebral palsy and a group of healthy pediatric patients

**DOI:** 10.4317/medoral.19568

**Published:** 2014-03-08

**Authors:** Alejandro Escanilla-Casal, Mirella Aznar-Gómez, José M. Viaño, Ana López-Giménez, Alejandro Rivera-Baró

**Affiliations:** 1 Adjunct in the Pediatric Dentistry and Orthodontics Service. Hospital Sant Joan de Déu Barcelona; 2 Resident in the Pediatric Dentistry and Orthodontics Service. Hospital Sant Joan de Déu Barcelona; 3 Head of the Pediatric Dentistry and Orthodontics Service. Hospital Sant Joan de Déu Barcelona

## Abstract

This is a comparative study between two groups, one of healthy children and the other of children with cerebral palsy, which underwent dental treatment under general anesthesia at Hospital Sant Joan de Déu Barcelona. The purpose of the study was to compare and determine oral pathology, frequency, severity and postoperative complications in pediatric patients with and without an underlying disease which undergo a dental treatment under general anesthesia.

** Key words:**General anesthesia, cerebral palsy, pediatric patients.

## Introduction

Cerebral palsy (CP) groups together permanent but non-progressive disorders that damage the motor control centers of the brain and affect the patient’s psychomotor activity,often accompanied by sensation, cognitive, communication and, occasionally, behavior problems. Clinical symptoms may change over time due to the interaction of pathological motor patterns associated with the maturation of the central nervous system (CNS) (1).

Caused by a variety of factors (prenatal [35%], gestational [55%] or postnatal [10%]), it can affect one or several motor areas of the CNS from the fetal period up to age 5, with a prevalence of between 2 and 2.5 cases per 1,000 births. It is diagnosed in all countries, affects all ethnic groups, and there is no clarity regarding gender-specific prevalence.

Although attempts have been made to classify it in many ways, the most useful today is by the location of the lesion and its symptoms:

a) Spastic CP: is the second most common clinical form and accounts for 50 to 75% of cases. The lesion is located in part of the cortex, including the pyramidal tract and other levels like the internal capsule, the midbrain and the spinal cord. In terms of motor activity, patients present muscular hypertonia, hyperreflexia, hyperflexion and difficulty with voluntary motor control.

b) Athetoid/dystonic CP: accounts for 15% of cases. The lesion is located in the basal ganglia and in the extrapyramidal nervous system. In terms of motor activity, patients present hypotonia at rest and hypertonia in response to stimulation, involuntary movements, twitching of limbs, and abnormal postures, and it may affect any part of the body (face, tongue, hands and feet).

c) Ataxic CP: is the least common form, accounts for 5 to 10% of cases and is mainly located in the cerebellum. It is characterized by alterations in body balance, generalized hypotonia and uncoordinated voluntary movements.

d) Mixed CP: is the most common form, can affect any part of the CNS and primarily manifests in spasticity in upper and lower limbs and dystonic/athetoidsymptoms in the body and head.

It should also be noted that conditions associated with CP include psychological impairment and mental retardation (30%), epilepsy (12-90%), hearing disorders (60%), vision disorders (23%), dysarthria, gastrointestinal and nutritional problems, urinary incontinence, and laryngeal and diaphragmatic dysfunctions (2).

With respect to oral manifestations, these patients have a higher rate of caries and bacterial plaque in baby and permanent teeth compared to the general population due to a variety of factors, including difficulty controlling oral hygiene, the soft diet they follow and the difficulties that many have in chewing and swallowing (3).

Also of note is the fact that these patients have an increased frequency of gingival hypertrophy, likely resulting from the decreased mechanical action of crushing food, and a greater presence of hypoplasia and injuries to upper front teeth, due to the increase in falls caused by lack of motor control. CP patients present a high percentage of bruxism, with some authors establishing it at between 40 and 70% of cases, primarily affecting the teeth of the upper jaw and the lower molars and premolars. As for drooling, it is attributed to poor coordination during the voluntary phase of swallowing. Lastly, the most common malocclusion in CP patients is Class II with open bite and overjet due to the muscular hyperextension of the head which causes stretching of the oral soft tissues that contributes to mandibular retrognathia and vertical growth, causing molars to over-erupt and favoringlow tongue position (4).

## Material and Methods

The study included a total of 86 patients treated under general anesthesia for dental reasons divided into two groups. Group 1 (n=48) was comprised of healthy children, 20 males (41.7%) and 28 females (58.3%), while group 2 (n=38) was comprised of patients with cerebral palsy, 20 males (52.6%) and 18 females (47.4%). The age for group 1 ranged between 2 and 16 years old with a mean age of 6.72 years, and the age for group 2 ranged between 2 and 19 years old with a mean age of 10.57 years.

All patients were treated at the Pediatric Dentistry and Orthodontics Service of the Hospital Universitario Materno infantil Sant Joan de Déu Barcelona between October 2010 and March 2011.

Inclusion criteria were patients diagnosed with cerebral palsy 18 years old or under and healthy patients with extensive oral health problems with difficult behavior management 4 years old or under who required dental treatments or oral surgery under general anesthesia.

We excluded patients referred from other centers and patients who did not submit the postoperative assessment questionnaire.

We obtained the standard informed written consent of the Hospital Sant Joan de Déu Barcelona from the patients’ legal guardians prior to the clinical examination, in accordance with the ethical principles of the World Medical Association agreed in the Declaration of Helsinki (version 2002).

A specific form was used to record the surgical data where we noted the qualitative and quantitative data of the treatments performed:types and number of treatments performed during the surgery, drugs (which included the drugs administered during the surgery), duration of general anesthesia, duration of the dental treatment and length of hospitalization.

Postoperative complications were recorded in the patient’s medical record by nurses in the immediate postoperative period. There was also a postoperative questionnaire during the first 72 hours following surgery, which was provided to parents the day patients were discharged from the hospital, and an interview in the post-surgical follow-up conducted at the Pediatric Dentistry and Orthodontics Service several days after the surgery.

The following variables were on the Pediatric Dentistry and Orthodontics Service’s postoperative assessment questionnaire:

-Dental pain (Mild: does not require pain reliever; Moderate: goes away with pain reliever; Severe: persists with pain reliever) 

-Cough (Mild: a few times/day; Moderate: several times/day; Severe: many times/day)

-Nausea (Mild:once/day; Moderate: twice/day; Severe: more than twice/day)

-Vomiting (Mild:once/day; Moderate: twice/day; Severe: more than twice/day)

-Fever (Mild: up to 37ºC; Moderate: up to 38ºC; Severe: over 38ºC)

-Bleeding wound in mouth

-Sore throat/neck

-Lack of appetite 

-Sleepiness/Drowsiness

-Insomnia

-Psychological changes:night terrors, nervousness, restlessness

-Other complications (type, duration)

The statistical analysis of the data was performed using the statistical package SPSS-PC 17.0.

We calculated the following descriptive parameters for each variable: mean, standard deviation, maximum and minimum.

The group of healthy children was compared to the group of children with cerebral palsy using Student’s t-test if the variables were quantitative and Pearson’s chi-squared test if the variables were qualitative. The level of significance used was 0.05.

## Results

 Table 1 shows the descriptive statistical data for the healthy group (H) and the cerebral palsy group (CP). The chi-squared test was used for intergroup comparison. The mean age in the CP group was higher, but there were no statistically significant differences between both groups in terms of the duration of general anesthesia, the duration of the dental treatment or the length of hospitalization. We did find significant differences (*p*<.05) in the type of admission, where ambulatory surgery was 75% more common in the H group than in the CP group ( Table 2). Moreover, we did not find intergroup differences in the pharmacological treatments used.

**Table 1:**
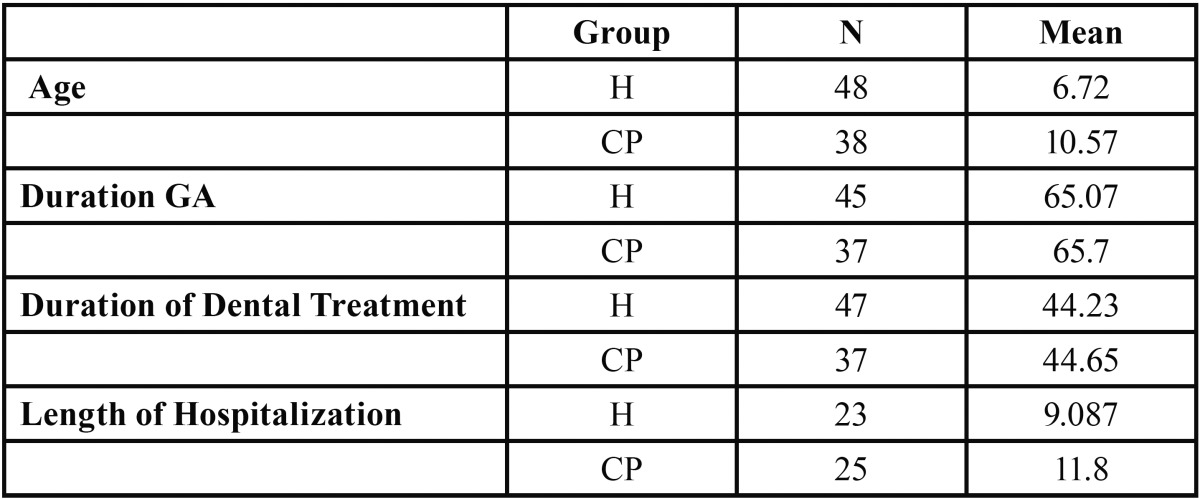
Descriptive Data of the H Group and the CP Group.

**Table 2:**
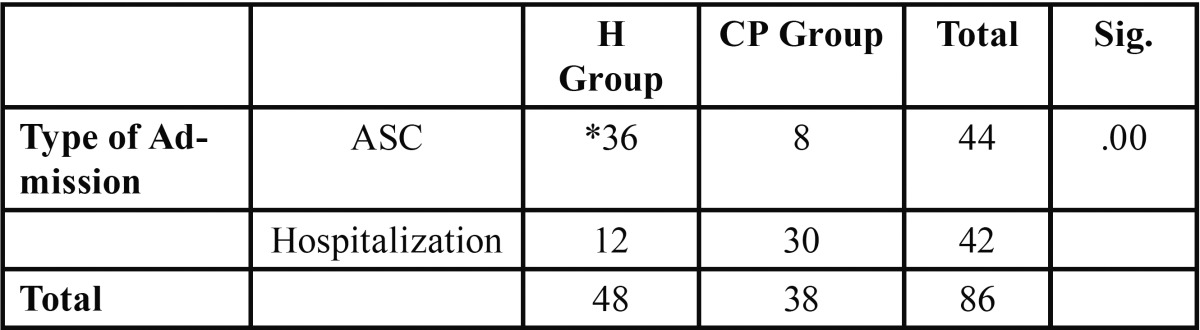
Type of Admission.Chi-squared Test.

With regard to treatments performed ( Table 3), pulpotomies were conducted more frequently in the group of healthy patients with a significance of *p*<.00. More teeth cleanings, fillings and extractions were performed on the CP group than on the H group(*p*<.05).

**Table 3:**
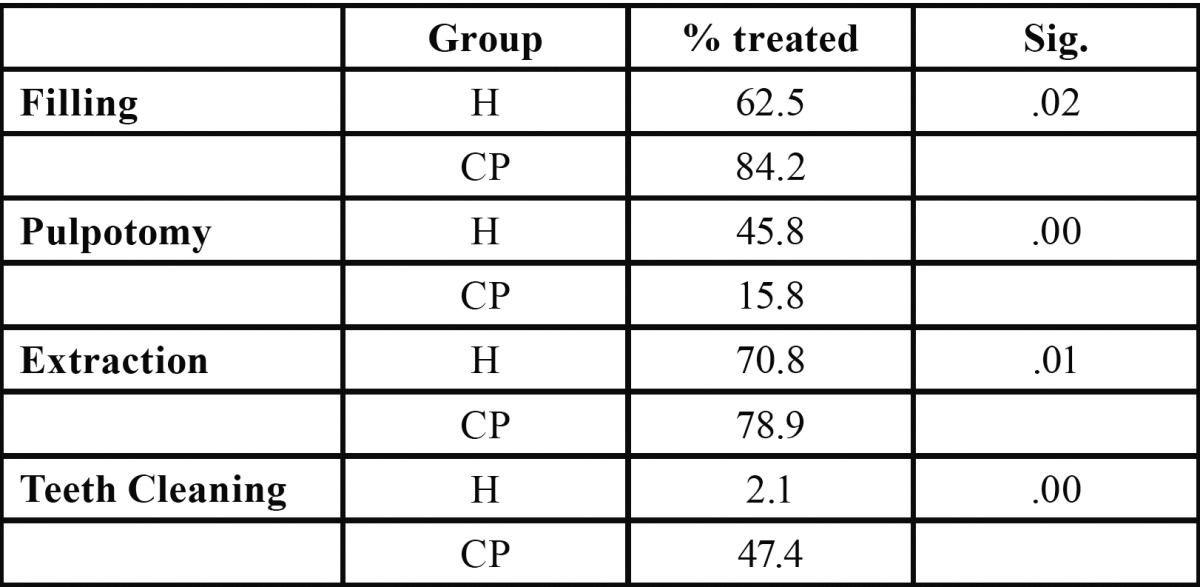
Treatments Performed on Both Groups, Using the Chi-squared Test.

As for postoperative complications recorded on the questionnaire after the first day, the CP group had more bleeding and more sleepiness (*p*<.05)( Table 4).

**Table 4:**
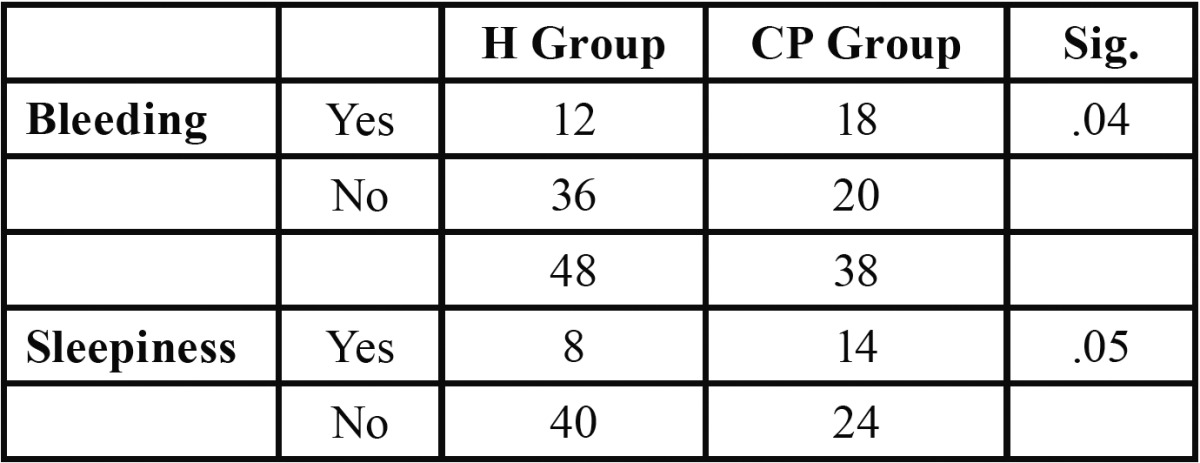
Complications Following the First Day after Surgery.

## Discussion

The purpose of this study was to identify and quantify different variables of dental treatment under general anesthesia (GA) in healthy pediatric patients (H) and pediatric patients with cerebral palsy (CP). This type of technique is useful for a particular population group, such as young patients who require extensive dental treatment and who do not achieve desired results with the use of local anesthesia and behavior management. The other population group suitable for such treatment is patients with some sort of physical/psychological disability who are often characterized by poor oral hygiene, soft and cariogenic diets, periodontal disease and polypharmacy (5,6). There is also the group of medically compromised patients who, due to this fact and to their underlying disease, must undergo surgery under GA so they may be monitored better.

Our study showed no significant differences regarding age distribution, where the mean age was 6 years 7 months in healthy patients and 10 years 5 months in cerebral palsy patients. These results are in line with a publication where the authors observed that special patients were older than healthy patients, specifically healthy patients were 4 years 2 months old and special patients were 5 years 5 months old (7,8). These findings are related to Hospital Sant Joan de Déu Barcelona’s admission criteria: to be eligible for dental treatment under GA, healthy patients with multiple caries must not be over 4 years old. Another factor to bear in mind which influences this age difference is that, due to their underlying disease, parents or caregivers of special patients view oral health as a matter of secondary importance.

We did not find significant differences between the H group and the CP group with respect to the duration of general anesthesia, with an average of 65.07 minutes for the former and 65.70 minutes for the latter. Our criterion is consistent with the recommendations of other authors, which had an average of 80 minutes (9), 30 minutes (10) and 124 minutes (11). According to these authors’ experience, the duration of GA in patients treated in ambulatory surgery centers (ASCs) should not exceed 120 minutes (9). In its guides, the Royal College of Surgeons of England proposes that the duration of general anesthesia in ASC patients should not exceed 30 to 40 minutes (10).

Regarding the length of hospital stay, we had more cerebral palsy patients admitted to the hospital and for a longer period of time than healthy patients. This can be explained by the fact that healthy patients meet criteria to be treated in ASCs and therefore the length of hospitalization is shorter. We obtained significant differences in the type of admission, where ambulatory surgery was 75% more common in the H group than in the CP group. Ambulatory surgery is an ideal solution for providing quality healthcare while decreasing socioeconomic costs. These advantages have been published in previous studies which have demonstrated that ASCs are highly useful for conducting dental treatments in certain population groups, as ambulatory surgery is safe and widely accepted by patients and parents alike, causing minimal social and family changes, and also reduces waiting times and costs (9,12). The results from our study also confirm these findings. Our criteria is consistent with other authors in recommending ASA I-II patients as candidates for ambulatory surgery, but advising against ASA III-IV patients for ambulatory surgery due to the complexity of their underlying disease.

The results related to the type of dental treatments showed a higher percentage of dental fillings (84.2%) in the CP group than in the H group (62.5%). These findings could be associated with the high prevalence of caries and an increased number of teeth present in the mouth in the CP group (32 teeth) versus the younger H group (20 teeth). However, more pulpotomies were conducted on the H group (45.8%) than on the CP group (15.8%), likely due to the fact that a pulpotomy is a treatment conducted on baby teeth, i.e., on young patients, as is the case of healthy patients under 4 years old (13,14). We found significant differences with respect to extractions, like other publications which found that ASA II-III patients underwent a greater number of extractions due to the more aggressive protocol followed for the group of patients with an underlying disease, since conservative dental treatments implicitly have a greater risk of failure, which is why extractions prevail in certain cases (13). We also found significant differences in teeth cleanings, which needed to be performed with greater frequency on the CP group. We associate this datum with the difficulty parents have in daily brushing and with these patients’ soft diets or because they have a gastric feeding tube. These factors result in poor chewing functions and cause a high incidence of supra and subgingival plaque (6).

With regard to complications following the first day after surgery, we found more bleeding and sleepiness in the CP group. The increased bleeding in this type of patient may be caused by the difficulty in carrying out proper hemostasis with gauze pressure following the surgery and by the constant drooling which often characterizes these patients. It should also be noted that more extractions were performed on this group than on the H group.

Some authors have observed a relationship between sleepiness and the duration of GA, where they found that for each additional 10 minutes in the duration of GA, alterations in sleep increased by 15% (11,15).

Lastly, more effort should be made to motivate these patients and their caregivers to visit the dentist and receive primary prevention. There should also be more fluid communication between pediatric dentists and pediatricians supported by specific programs to prevent oral health problems.
